# Pro-Inflammatory Cytokines Predict Relapse-Free Survival after One Month of Interferon-α but Not Observation in Intermediate Risk Melanoma Patients

**DOI:** 10.1371/journal.pone.0132745

**Published:** 2015-07-20

**Authors:** Ahmad A. Tarhini, Yan Lin, Haris Zahoor, Yongli Shuai, Lisa H. Butterfield, Steven Ringquist, Helen Gogas, Cindy Sander, Sandra Lee, Sanjiv S. Agarwala, John M. Kirwood

**Affiliations:** 1 University of Pittsburgh, Pittsburgh, Pennsylvania, United States of America; 2 University of Pittsburgh Cancer Institute Biostatistics Facility, Pittsburgh, Pennsylvania, United States of America; 3 Hellenic Cooperative Oncology Group, Athens, Greece; 4 Dana Farber Cancer Institute, Boston, Massachusetts, United States of America; 5 St. Luke's Cancer Center, Bethlehem, Pennsylvania, United States of America; 6 Temple University, Philadelphia, Pennsylvania, United States of America; Rutgers University, UNITED STATES

## Abstract

**Background:**

E1697 was a phase III trial of adjuvant interferon (IFN)-α2b for one month (Arm B) versus observation (Arm A) in patients with resected melanoma at intermediate risk. We evaluated the levels of candidate serum cytokines, the HLA genotype, polymorphisms of CTLA4 and FOXP3 genes and the development of autoantibodies for their association with relapse free survival (RFS) in Arm A and Arm B among 268 patients with banked biospecimens.

**Methods:**

ELISA was used to test 5 autoantibodies. Luminex/One Lambda LABTypeRSSO was used for HLA Genotyping. Selected *CTLA4* and *FOXP3* Single nucleotide polymorphisms (SNPs) and microsatellites were tested for by polymerase chain reaction (PCR). Sixteen serum cytokines were tested at baseline and one month by Luminex xMAP multiplex technology. Cox Proportional Hazards model was applied and the Wald test was used to test the marginal association of each individual marker and RFS. We used the Lasso approach to select the markers to be included in a multi-marker Cox Proportional Hazards model. The ability of the resulting models to predict one year RFS was evaluated by the time-dependent ROC curve. The leave-one-out method of cross validation (LOOCV) was used to avoid over-fitting of the data.

**Results:**

In the multi-marker modeling analysis conducted in Arm B, one month serum IL2Rα, IL-12p40 and IFNα levels predicted one year RFS with LOOCV AUC = 82%. Among the three markers selected, IL2Rα and IFNα were the most stable (selected in all the cross validation cycles). The risk score (linear combination of the 3 markers) separated the RFS curves of low and high risk groups well (p = 0.05). This model did not hold for Arm A, indicating a differential marker profile in Arm B linked to the intervention (adjuvant therapy).

**Conclusions:**

Early on-treatment proinflammatory serum markers (IL2Rα, IL-12p40, IFNα) significantly predict RFS in our cohort of patients treated with adjuvant IFN-α2b and warrant further study.

## Introduction

Host immunity plays a key role in tumor surveillance and can result in a cell-mediated proinflammatory response to cancer and tumor suppression, or tolerance and tumor progression.[1,2] As the tumor-immune cell cross-talk evolves, modulation between inflammation and immunosuppression within the tumor microenvironment ultimately results in tumor elimination, resistance or immune tolerance.[3] Melanoma is a tumor that has proven to be responsive to immunity both in the adjuvant and advanced disease settings. Reports of spontaneous tumor regression first suggested a role for host immunity in melanoma, which was also supported by the frequent observation of lymphoid infiltrates at the primary melanoma site, and histological signs of tumor regression. Host cellular immune response within melanoma tumor tissue has potential prognostic and further predictive significance in relation to the likelihood of response to immunotherapies.[4] T cell infiltrates in primary melanoma are prognostic of disease outcome,[5] and T cell infiltrates within regional nodal metastasis predict benefit from neoadjuvant IFN-2b therapy.[6–8] This characteristic of melanoma has been exploited to develop several immunotherapeutic regimens that have significantly impacted the management of this disease.[9–12]

E1697 was a phase III trial that studied the impact of a 4-week course of high dose interferon (IFN)-α2b given intravenously versus observation in patients with resected melanoma of intermediate risk. This trial was terminated as recommended by the Eastern Cooperative Oncology Group’s Data Monitoring Committee after a third interim analysis that found no evidence of durable benefit from treatment with IFNα-2b.[13] This outcome made it essential to investigate biomarkers of therapeutic predictive value in this study population that may still allow the identification of a subpopulation that had clinical benefit. Identification of significant predictive markers may have clinical implications and help guide the design of future adjuvant trials.

Defining biomarkers in the peripheral blood is of particular interest given the accessibility of the biospecimen and the relative ease of testing. Therefore, we evaluated the levels of candidate serum cytokines individually selected based on prior studies for their immunotherapeutic predictive or prognostic value in melanoma.[14–18] In addition, we tested the prognostic value of the development of autoimmunity induced by IFN-α2b. This is based on an association reported between the development of autoimmunity and favorable antitumor effects for several forms of immunotherapy including IFN-α2b, Interleukin (IL)-2 and anti-CTLA4 blocking antibodies among patients with melanoma [9–20]. Since autoimmunity induced by IFN-α occurs during the course of therapy and cannot be used as a baseline predictive biomarker,[21] we were interested in evaluating the potential baseline predictors of the risk of autoimmunity in this trial. Therefore, we tested genetic predisposition to the induction of autoimmunity as a potential prognostic factor by evaluating the HLA genotypes and selected polymorphisms in cytotoxic T-lymphocyte-associated protein 4 (CTLA-4) and FOXP3 genes.[20,22]

In this study, we hypothesized that multi-marker modeling analysis of the selected markers may generate a prognostic biomarker signature at baseline or early on-treatment (at one month of IFN-α-2b) biomarker signature in this patient population. In melanoma adjuvant IFN-α studies, relapse free survival (RFS) has been the most consistent and reproducible efficacy endpoint across multiple trials.[23,24] Therefore, we used RFS as our primary efficacy endpoint for this analysis.

## Materials and Methods

### Study design and patients

Eastern Cooperative Oncology Group (ECOG)–led U.S. Intergroup trial E1697 compared one month of adjuvant IFNα-2b therapy (Arm B) with observation alone (Arm A) for patients with resected melanoma at intermediate risk of relapse and death (T2b N0, T3a-b N0, T4a-b N0, T1-4 N1-2a). Patients in the intervention arm (Arm B) received IFNα-2b intravenously 5 days a week for a total 4 weeks (20 doses). We used banked serum and lymphocyte samples from 268 patients enrolled in the trial for the biomarker testing. The original clinical study (U.S. Intergroup E1697) and this correlative study were approved by the Eastern Cooperative Oncology Group, the Cancer Therapeutic Evaluation Program of the National Cancer Institute, and Institutional Review Board (IRB) responsible for each treating institution. Written informed consent was obtained from study participants or a legally authorized representative prior to enrollment. The IRB at the University of Pittsburgh where the laboratory correlative studies were conducted approved the study and consent.

### Procedures

Using standardized phlebotomy procedures, peripheral blood was drawn from each of the patients in blood tubes provided to each clinical site by the ECOG Central Immunology Lab at the UPCI Immunologic Monitoring and Cellular Products Laboratory (IMCPL), and shipped overnight back to the laboratory in insulated containers. Blood was processed upon receipt. Samples utilized in this study were obtained from subjects after study enrollment but prior to treatment initiation (baseline), after one month, then at 3, 6, 9 and 12 months. Peripheral blood mononuclear cells (PBMC) were isolated from heparin-containing tubes by a Ficoll gradient centrifugation and cryopreserved according to standard operating procedures (SOP) and stored in continuously monitored freezers. Blood samples were collected without anticoagulant into red top vacutainers and allowed to coagulate for 20–30 minutes at room temperature. Sera were separated by centrifugation according to SOPs, and all specimens were immediately aliquoted, frozen and stored in a monitored –80°C freezer. No freeze-thaw cycles were allowed before testing for each sample.[25]

The xMAP Luminex serum assay for the selected cytokines (FGF-basic, IL-2, IL-2R, IL-6, IL-8, IL-10, IL-12p40, IL-17, TNF-α, IFN-α, MIP-1α, MIP-1β, IP-10, VEGF, IL-1β, IL-1α) was performed, utilizing baseline and one month specimens, according to the manufacturer’s protocol (BioSource International (Camarillo, CA)) as previously described,[26] using a kit pre-tested for antibody cross-reactivity and analyzed on the Bio-Plex suspension array system (Bio-Rad Laboratories, Hercules, CA).[26] C-reactive Protein (CRP) was run singly as it requires different dilutions. Controls included kit provided standards, and the Multiplex cytokine QC mix (R&D Systems).[26]

For the detection of autoantibodies, sera were tested at baseline, and then at 1, 3, 6, 9 and 12 months using a qualitative enzyme-linked immunosorbent assay (DIASTAT; Euro-Diagnostica, Malmo, Sweden) following the manufacturer’s protocol. Serum samples were tested for the presence of the following autoantibodies: antinuclear antibody screen (ANA) [positive absorbance ratio (sample absorbance value/mean reference control absorbance value) ≥ 1.0], antithyroglobulin antibody (TG) (positive > 1.0), antithyroperoxidase antibody (TPO) (positive > 1.0), and anticardiolipin antibody (TACL) (total: IgA + IgM + IgG; positive concentration >21 PL U/ml).

HLA Genotyping was conducted with Luminex/One Lambda LABTypeRSSO. PCR was used to test CTLA4 and FOXP3 SNPs and microsatellites. The CTLA-4 gene has been mapped to chromosome 2q33.3 and consists of 4 exons.[27] We tested the three most frequently studied polymorphisms; a dinucleotide repeat in the 3’ untranslated region (VNTR), a G/A transition in exon 1 at position +49 and a C/T transition (CT60) within the 3’-untranslated region.[28] The Foxp3 gene is located on chromosome Xp11.23.[29] We tested a microsatellite functional polymorphism (GT)n in the promoter/enhancer region where variant expression has been implicated in dysregulation of T cells leading to autoimmune diseases.[29]

### Statistical analysis

All statistical analysis was conducted using SAS9.3 (SAS Institute, Cary NC) and R 3.1.3 (http://www.r-project.org). Univariate proportional hazard (PH) models were used to assess the association between each marker and RFS. The Benjamini and Hochberg method was used to adjust for multiple testing. Markers with detection rate < 70% were dichotomized as detected versus not detected. In the process of multi-marker prognostic model building, to avoid over fitting, leave-one-out cross validation (LOOCV) was used to evaluate the performance of the multi-marker model. Within each LOOCV cycle, the least absolute shrinkage and selection operator (Lasso) approach, implemented in the glmnet package, was used to select combination markers that are most informative for RFS in the training data.[30] Markers with coefficients ≥ 0.1 in the Lasso CoxPH model were selected and included in a regular CoxPH model, which is then used to generate the risk score of the leave-out sample. The survival receiver operating characteristic (ROC) analysis was used to evaluate the ability of the models to predict 1-year RFS.[31] In conducting a true cross validation within each LOOCV cycle, the selected markers were different due to fluctuation of the data. The area under the ROC curve (AUROC) is the product of this cross validation procedure. The top markers that are selected in over 90% of the cycles are considered in the final signature.

We calculated the final RS using the three top markers by fitting a CoxPH model, *h*(*t*) = *h*_0_(*t*)exp(*β*_1_ × *IL*2*Rα* + *β*_2_ × *IL*-12*p*40 + *β*_3_ *IFNα*) to the whole dataset.

The RS was calculated as the linear combination of the model as the following *RS* = *β*_1_ × *IL*2*Rα* + *β*_2_ × *IL*-12*p*40 + *β*_3_ *IFNα*

We then divided the patients into two risk groups, the high risk group with RS >median (RS), and the low risk group with RS< = median (RS). The log rank test was used to compare the RFS of the two risk groups. Univariate analysis and multivariate CoxPH model were used to evaluate the association of the clinical factors and RS to RFS.

## Results

### Single marker association with RFS

Table 1 summarizes the patient and disease characteristics of the included patients. We tested the association of the level of each marker at baseline and early on treatment (one month) with RFS within each treatment arm. Controlling the false discovery rate (FDR) at 20%, we noticed that the levels of certain markers have differential effects between the two arms. For example, the level of IFNα at 1 month was only prognostic in Arm B (adjuvant IFN-α2b) but not in Arm A (observation group). Although under-powered, we explored the potential predictive value of each marker by testing the interaction between the marker level and the treatment effects. We noticed a trend toward significance for IFNα at both baseline and one month post treatment (unadjusted p-value <0.05). Other markers that achieved a p-value <0.05 included IL-10 at baseline and HLA-B-44 at one month post treatment. Results of the univariate analyses are included in S1 File. Results of the marker level- treatment effect interaction tests are included in S2 File.

**Table 1:** Patient and disease characteristics (N = 216).

	Arm A (Observation)		Arm B (IFN)	
	No. of Patients (N = 105)	%	No. of Patients (N = 111)	%
**Sex**				
Male	53	24.5	61	28.2
Female	52	24.1	50	23.2
**Age**				
>50	62	28.7	66	30.6
≤50	43	19.9	45	20.8
**Breslow thickness**				
≤0.75mm	1	0.5	2	0.9
0.76–1.5 mm	16	7.6	17	8
1.51–4.0 mm	72	34	70	33
≥4.1 mm	15	7.1	19	8.9
**Ulceration**				
Yes	53	24.5	49	22.7
No	50	23	60	28
Unknown	2	0.9	2	0.9
**Clark’s level of invasion**				
1	8	3.7	4	1.9
2	2	0.9	3	1.4
3	11	5.1	9	4.2
4	70	32.4	85	39.4
5	13	6	10	4.6
Unknown	1	0.5	-	-
**1-year RFS**				
Free	81	37.5	84	38.9
Relapse	18	8.3	21	9.7
Unknown	6	2.8	6	2.8

### Multi-marker prognostic models

**Fig 1:**
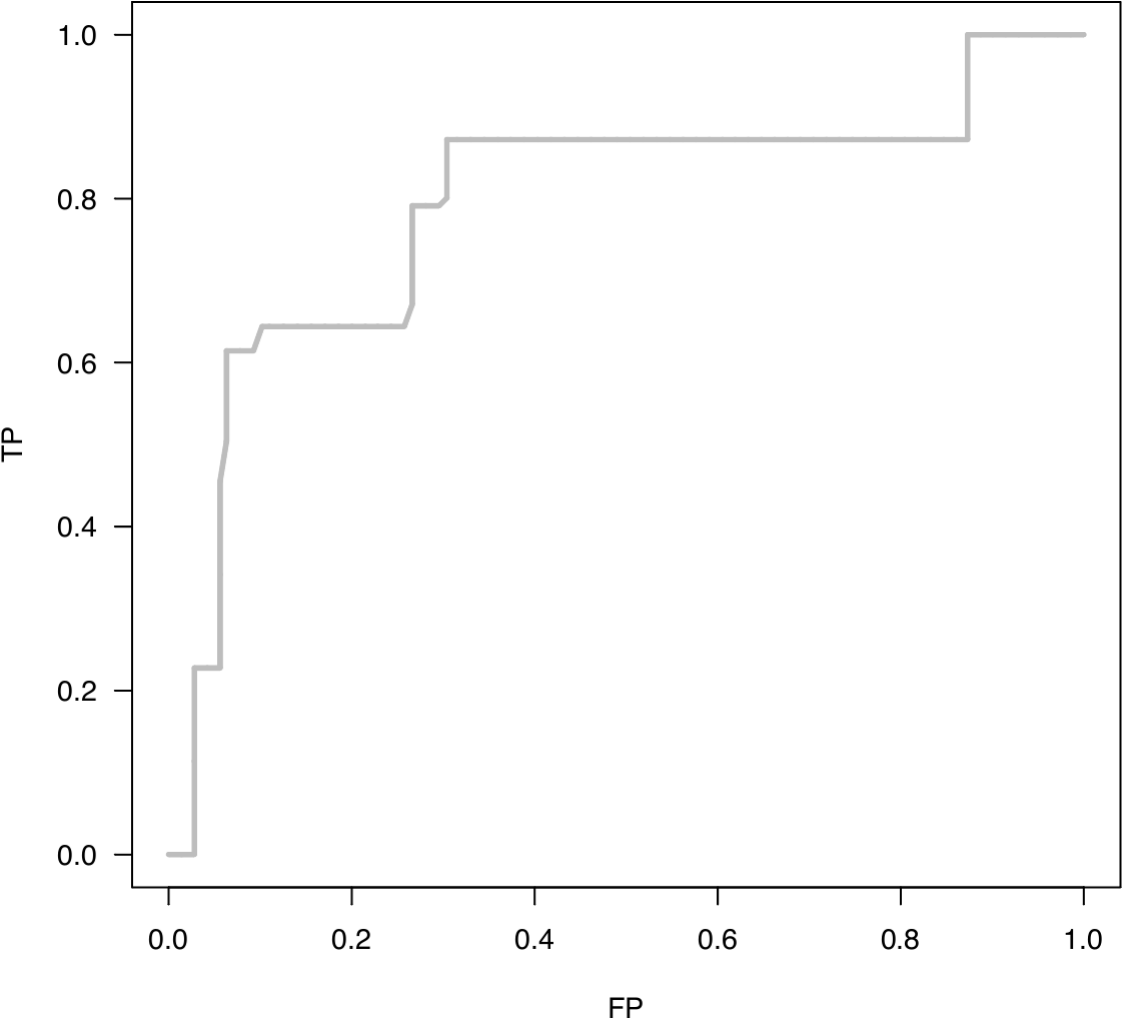
Receiver operating characteristic (ROC) survival analysis in Arm B (patients treated with one month of interferon-α). The levels of pro-inflammatory cytokines (IL2Rα, IL-12p40 and IFN-α) at one month predict one year relapse free survival (RFS). Dichotomizing the linear multi-marker risk score at the median, the patients were separated as high and low risk groups. The leave-one-out cross validation (LOOCV) AUC was 82%. TP: true positive rate. FP: false positive rate.

**Fig 2:**
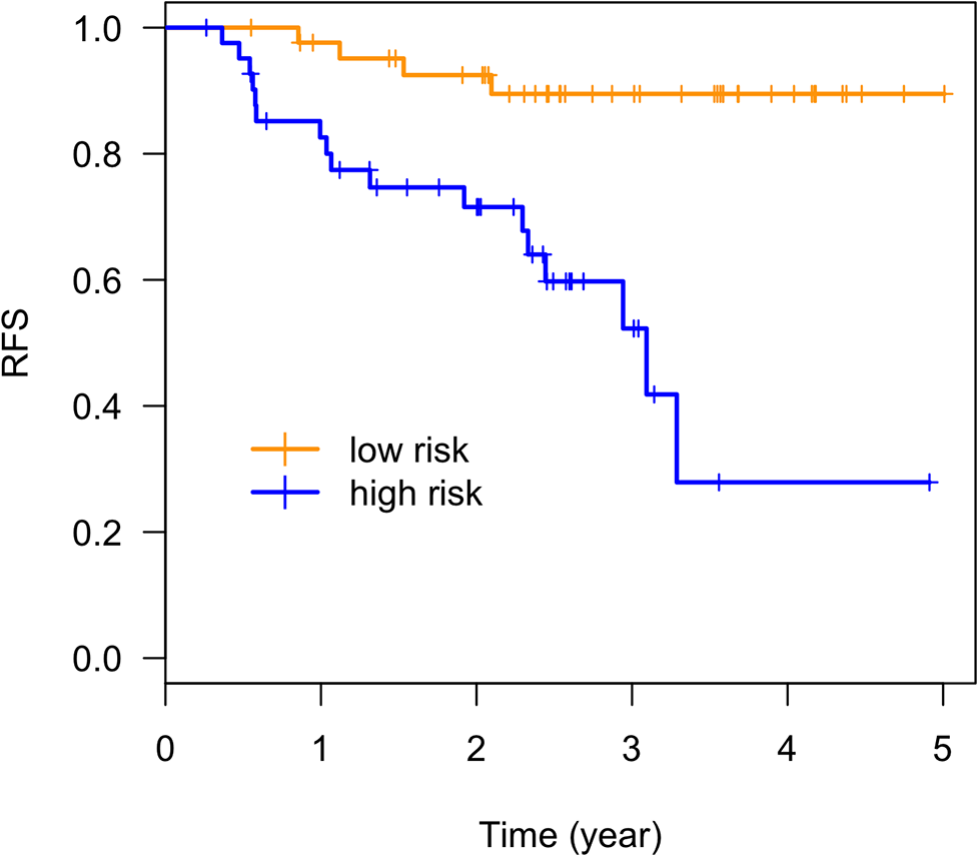
The Kaplan–Meier (KM) plot of RFS by the dichotomized multi-marker score in Arm B (patients treated with one month of interferon-α). One month pro-inflammatory cytokines (IL2Rα, IL-12p40 and IFN-α) predict one year RFS.

We attempted to build a multi-maker prognostic model for each treatment arm using baseline and one month maker data. LOOCV was used in the evaluation. Within each cycle of the cross validation, we used the Lasso approach to select the combination of markers that were associated with RFS of the patients in each study arm and build a CoxPH model using the training dataset. This model was then used to generate a risk score for the leave out sample. This process was repeated until all samples were left out once. The survival receiver operating characteristic (ROC) analysis[31] was then applied to the resulting predicted risk score for each patient to evaluate the ability of the model in the prediction of one year RFS. Using this approach, we discovered one panel of markers circulating cytokines, IL2Rα, IL-12p40 and IFNα that significantly predicted one year RFS at one month post adjuvant IFN-α2b treatment in Arm B. The LOOCV AUC was 82%. Fig 1 illustrates the model prediction of one year RFS using survival ROC analysis in Arm B. A three-marker risk score (RS) is calculated based on a CoxPH model of these three markers as described in the methods. Dichotomizing the RS at the median, the patients in Arm B were separated as high and low risk groups. Fig 2 presents the Kaplan–Meier (KM) plot of RFS of the two groups. The RFS of the high and low risk groups defined by the three marker RS were significantly different (p-value = 0.002). This model did not hold for Arm A (Observation), indicating differential roles marker profiles in Arms A and B. These results are consistent with what we have determined in the univariate analysis.

We evaluated the clinical factor in relation to RFS by Univariate CoxPH model. Using data from both arms A and B, we observed that factors that are marginally associated with RFS in our data were Breslow’s thickness, ulceration, sex and age (see the univariate analysis results in the following table). Upon further investigation we observed that age and sex were highly correlated (women were significantly younger than man, p<0.001), and the final best multivariate clinical model included Breslow’s thickness, ulceration, and age (see the multivariate analysis results in **Table 2**). When we included the clinical factors and the dichotomized risk score (RS) in the same multivariate CoxPH model, the RS remained significantly associated with RFS (p = 0.0024). Therefore, the three-marker signature was found to be significantly associated with RFS, independent of the clinical factors. Therefore, the RS may potentially improve the prediction of the prognosis of the patients under study.

**Table 2:** The association of clinical factors with relapse free survival (RFS).

	Univariate Analysis		Multivariate Analysis	
Clinical Variable	HR	P-value	HR	P-value
Treatment Arm	0.982	0.947		
sex	0.568	0.046		
**Breslow Thickness**	1.127	0.015	1.079	0.131
Clark Level	1.211	0.168		
LDH_RS	0.999	0.409		
LDH_ULN	0.998	0.168		
Pigmentation	1.117	0.317		
PS score	0.963	0.931		
**Ulceration**	1.836	0.020	1.613	0.062
**Age**	1.034	0.005	1.028	0.021

## Discussion

Our selection of the panel of candidate cytokines to be tested in this study was data-driven from previous reports by our group and others.[14–18] In the context of the E1694 trial, we previously reported that 3 months post initiation of IFNα therapy serum levels of angiogenic and growth factors including VEGF, EGF and HGF were significantly decreased, whereas expression of IP-10, IFNα, MCP-1, IL-12p40, soluble TNF-RI, TNF-RII, and IL-2R were significantly increased.[14] E1694 tested high dose IFN-α given for one year as compared to the ganglioside GMK vaccine in a high-risk resected melanoma population and demonstrated significant RFS and OS benefit in favor of IFNα. High baseline proinflammatory cytokine levels predicted RFS benefit in the IFNα treatment group, but not the control GMK vaccine group.[25] This is consistent with our present analysis conducted in the context of the one month IFNα trial E1697, where we sought to test the predictive value of the proinflammatory cytokine profile, except that the current analysis has shown the value of these changes early on-treatment (after one month of IFNα). In E1697, IFNα was given for only one month and the treated patients had a lower disease recurrence risk profile than the E1694 population. E1697 is a negative study in relation to its impact on RFS overall while E1694 demonstrated significant benefits in the population and regimen tested. Unfortunately, E1694 did not collect biospecimens at one month of IFNα therapy and therefore, it has not been possible to test biomarkers at the one month time point from that study. The proinflammatory immune response generated after one month of IFNα therapy is consistent with its immunomodulatory impact on the host immune response.[32] In fact, immunologic modulation has been proposed as the most likely mechanism for the adjuvant benefit of IFNα.[8,32]

In this study, the multimarker proinflammatory signature we have identified consisted of IL-2Rα, IL-12p40 and IFNα. Both univariate association tests and Lasso selection testing yielded the same set of markers at one month for Arm B. These markers had differential effects between arms B and A, indicating a potential predictive value. However, our study’s limited sample size restricts us from reaching a conclusive statement on the predictive value of the signature generated, but rather makes our results hypothesis generating due to our inability to conduct multiple testing adjustment. However, our results are particularly interesting when taken into context with previous reports identifying similar circulating analytes in the literature.[14] The potential prognostic value of the three markers identified here needs to be validated in a separate population.

The proinflammatory cytokines we identified have well-characterized immune regulatory functions. IFNα has distinct immunomodulatory actions that may provide an important link between the innate and adaptive immune responses.[33] In addition, it has direct effects on tumor cells inhibiting the proliferation and upregulating the expression of MHC class I antigens and adhesion molecules like ICAM-1 and l-selectin.[34,35] Tumor-induced angiogenesis is also inhibited by IFNα.[36] Soluble IL-2R is derived from a membrane receptor for IL-2, which is expressed on the cell surface of different lymphoid cell lines including activated T and NK cells, [37–39] and on some tumor cells.[40–42] It is comprised of three different chains: alpha (IL-2R*α*), beta (IL-2R*β*), and gamma chains (IL-2R*γ*). Tumor growth stimulates an immune response and increases IL-2R expression on immune cells and it’s shedding into the circulation.[43] Previous studies have indicated the potential utility of sIL-2R levels in clinical monitoring benefit from surgery and chemotherapy in patients with cancer.[44] IL-2R has been found to correlate with disease progression in melanoma,[45] and in patients with metastatic melanoma, was associated with tumor burden.[46] Lastly, IL-12p40 is a subunit of IL-12p70, a heterodimeric cytokine (with p35), that can be secreted by dendritic cells, and which can induce and skew immune response by promoting IFN-γ production and cytolytic activity of natural killer and T cells.[47] IL-12 also exhibits anti-angiogenic properties as it inhibited growth factor-induced corneal neovascularization in mice.[48] IFN-γ and NK cells are considered to be important effectors of anti-angiogenic properties of IL-12.[49,50] For these properties, IL-12 has been evaluated in the treatment of various cancers but with limited efficacy as tested to date.[47] IL-12p40 can also homodimerize, hence, specific measurement of the p70 heterodimer may clarify the functional role of increased IL-12p40 levels. Interestingly, a novel sequence termed p19 which shows no biological activity by itself may combine with the p40 subunit of IL-12 to form a novel, biologically active, composite cytokine, termed IL-23. IL-23 may have antitumor effect, stimulate IFN-γ production and proliferation of T cells as well as CD45RO (memory) T cells.[51]

The proinflammatory nature of the predictive cytokine profile found in our analysis is consistent with reported gene expression profiling studies of the tumor microenvironment (TME) in melanoma. Studies of the TME have supported the importance of the proinflammatory/inflamed microenvironment as a potential predictor of the benefit of immunotherapy. In melanoma metastases, the presence of lymphocytes correlated with the expression of defined chemokine genes, and a subset of 6 chemokines (CCL2, CCL3, CCL4, CCL5, CXCL9, and CXCL10) was confirmed by protein array and/or quantitative reverse transcription-PCR to be preferentially expressed in tumors that contained T cells.[52] A proinflammatory gene expression signature was reported to be associated with survival benefit following immunization with recombinant MAGE-A3 protein vaccine in patients with unresectable stage III or stage IV M1a metastatic melanoma.[53] Further, a pro-inflammatory/immune-reactive tumor microenvironment favored clinical response to ipilimumab and high dose interleukin-2.[54–56] Studies of immune cell infiltrates in melanoma have reported results consistent with this construct. Diffuse immune cell infiltrate throughout a metastatic tumor correlated best with survival in patients with metastatic melanoma. Higher densities of CD8+ T cells in the tumor microenvironment were the best predictor of improved survival.[4] Taken together with our proinflammatory cytokine data, these observations support the testing of baseline and /or early-on treatment biomarkers of the pro-inflammatory immune response in both tumor tissue and in circulating blood and evaluated simultaneously due to the common systems biology. Such parallel testing has the potential of generating a predictive biomarker signature in relation to IFNα and other immunotherapeutics such as ipilimumab or interleukin-2.

In our study population we have not found a prognostic role for the biomarkers associated with the induction of autoimmunity after one month of adjuvant IFNα. Similarly, no significantly prognostic markers were identified in the evaluation of the HLA genotypes and polymorphisms in CTLA4 and FOXP3. This is at variance with previous studies by our group and others.[21,57,58] However, key differences in the current study include the adjuvant IFNα regimen (one month versus one year) and the patient population under study (intermediate versus high risk). Therefore, additional investigations of these factors are warranted. As part of an ongoing scientific collaboration in the context of therapeutic and toxicity predictive value related to IFNα in E1697 we have completed our testing of IRF-5 polymorphisms and high-throughput SNP analysis of a wide array of genes with previously documented immunologic roles (Immunochip) to evaluate potential predictors of autoimmunity that may be associated with immunotherapeutic benefit. Biostatistical analysis of this work is ongoing at this time.

## Conclusion

Our modeling analysis in the context of the E1697 trial has generated a signature of three circulating pro-inflammatory serum biomarkers (IL-2Rα, IL-12p40, IFNα) that significantly predict RFS benefit. These early on-treatment serum biomarkers tested after one month of IFNα therapy significantly predicted RFS of patients treated with adjuvant IFNα. The model held only for the IFNα treatment group and not for the observation group, indicating a marker-treatment interaction. These data are consistent with our previously published predictive role of a proinflammatory cytokine profile in relation to IFNα therapeutic benefit and warrant further study in relation to IFNα and other immunotherapies of melanoma.

## Supporting Information

S1 File Univariate Cox Proportional Hazard Model analysis of each marker and relapse free survival (RFS). The xMAP Luminex serum assay for the selected cytokines (FGF-basic, IL-2, IL-2R, IL-6, IL-8, IL-10, IL-12p40, IL-17, TNF-α, IFN-α, MIP-1α, MIP-1β, IP-10, VEGF, IL-1β, IL-1α) was performed, utilizing baseline and one month specimens. CRP was run singly. Serum samples were tested for the presence of the following autoantibodies: antinuclear antibody screen (ANA), antithyroglobulin antibody (TG), antithyroperoxidase antibody (TPO), and anticardiolipin antibody (TACL). HLA Genotyping was conducted with Luminex/One Lambda LABTypeRSSO. PCR was used to test CTLA4 polymorphisms (AG49, CT60) and FOXP3 SNPs and microsatellites. Table A in S1 File shows the “Baseline” analysis and Table B in S1 File the “On-study (one month)” analysis.
(DOCX)

S2 File Testing of the predictive value of each marker. The xMAP Luminex serum assay for the selected cytokines (FGF-basic, IL-2, IL-2R, IL-6, IL-8, IL-10, IL-12p40, IL-17, TNF-α, IFN-α, MIP-1α, MIP-1β, IP-10, VEGF, IL-1β, IL-1α) was performed, utilizing baseline and one month specimens. CRP was run singly. Serum samples were tested for the presence of the following autoantibodies: antinuclear antibody screen (ANA), antithyroglobulin antibody (TG), antithyroperoxidase antibody (TPO), and anticardiolipin antibody (TACL). HLA Genotyping was conducted with Luminex/One Lambda LABTypeRSSO. PCR was used to test CTLA4 polymorphisms (AG49, CT60) and FOXP3 SNPs and microsatellites. Table A in S2 File shows the “Baseline” markers and Table B in S2 File the “On-study (one month)” markers.
(DOCX)
