# Supplementary material for: Pro-Inflammatory Cytokines Predict Relapse-Free Survival after One Month of Interferon-α but Not Observation in Intermediate Risk Melanoma Patients
Source: PLoS One. 2015 Jul 20;10(7):e0132745. doi: 10.1371/journal.pone.0132745 (PMC4508028; doi:10.1371/journal.pone.0132745)
Supplement: S1 File — The xMAP Luminex serum assay for the selected cytokines (FGF-basic, IL-2, IL-2R, IL-6, IL-8, IL-10, IL-12p40, IL-17, TNF-α, IFN-α, MIP-1α, MIP-1β, IP-10, VEGF, IL-1β, IL-1α) was performed, utilizing baseline and one month specimens. CRP was run singly. Serum samples were tested for the presence of the following autoantibodies: antinuclear antibody screen (ANA), antithyroglobulin antibody (TG), antithyroperoxidase antibody (TPO), and anticardiolipin antibody (TACL). HLA Genotyping was conducted with Luminex/One Lambda LABTypeRSSO. PCR was used to test CTLA4 polymorphisms (AG49, CT60) and FOXP3 SNPs and microsatellites. Table A in S1 File shows the “Baseline” analysis and Table B in S1 File the “On-study (one month)” analysis. (DOCX) [file pone.0132745.s001.docx]

**Supplementary Table 1. Univariate Cox Proportional Hazard Model analysis of each marker and relapse free survival (RFS). The xMAP Luminex serum assay for the selected cytokines (FGF-basic, IL-2, IL-2R, IL-6, IL-8, IL-10, IL-12p40, IL-17, TNF-α, IFN-α, MIP-1α, MIP-1β, IP-10, VEGF, IL-1β, IL-1α) was performed, utilizing baseline and one month specimens. CRP was run singly. Serum samples were tested for the presence of the following autoantibodies: antinuclear antibody screen (ANA), antithyroglobulin antibody (TG), antithyroperoxidase antibody (TPO), and anticardiolipin antibody (TACL). HLA Genotyping was conducted with Luminex™/One Lambda LABType™RSSO. PCR was used to test CTLA4 polymorphisms (AG49, CT60) and FOXP3 SNPs and microsatellites.**

**Table 1A.** Baseline analysis

| **Biomarkers** | **Treatment A** | | | **Treatment B** | | |
| --- | --- | --- | --- | --- | --- | --- |
|  | **P-value*** | **Adjusted P-value‡** | **Hazard Ratio #** | **P-value*** | **Adjusted P-value‡** | **Hazard Ratio#** |
| FGF_basic | 0.441 | 1.000 | 0.708 | 0.177 | 0.988 | 0.550 |
| Hu_IL2 | 0.986 | 1.000 | 1.009 | 0.071 | 0.988 | 0.321 |
| Hu_IL2R | 0.442 | 1.000 | 1.000 | 0.446 | 0.993 | 1.000 |
| Hu_IL6 | 0.466 | 1.000 | 1.000 | 0.829 | 0.993 | 1.000 |
| Hu_IL8 | 0.452 | 1.000 | 1.000 | 0.978 | 0.993 | 1.000 |
| Hu_IL10 | 0.092 | 1.000 | 2.260 | 0.158 | 0.988 | 0.346 |
| Hu_IL12 | 0.342 | 1.000 | 0.997 | 0.690 | 0.993 | 0.999 |
| Hu_IL17 | 0.359 | 1.000 | 1.531 | 0.594 | 0.993 | 1.253 |
| Hu_TNF | 0.832 | 1.000 | 1.109 | 0.989 | 0.993 | 1.006 |
| Hu_IFN | 0.208 | 1.000 | 1.745 | 0.101 | 0.988 | 0.454 |
| Hu_MIP_1a | 0.600 | 1.000 | 1.000 | 0.844 | 0.993 | 1.000 |
| Hu_MIP_1B | 0.533 | 1.000 | 1.000 | 0.793 | 0.993 | 1.000 |
| Hu_IP_10 | 0.287 | 1.000 | 0.984 | 0.523 | 0.993 | 0.996 |
| VEGF | 0.568 | 1.000 | 0.992 | 0.672 | 0.993 | 1.004 |
| Hu_IL_1B | 0.927 | 1.000 | 1.000 | 0.538 | 0.993 | 1.000 |
| IL_1_a | 0.588 | 1.000 | 1.000 | 0.383 | 0.993 | 0.998 |
| S100 | 0.663 | 1.000 | 0.125 | 0.834 | 0.993 | 0.252 |
| CRP, p | 0.883 | 1.000 | 1.000 | 0.216 | 0.993 | 1.000 |
| ANA | 0.210 | 1.000 | 0.000 | 0.820 | 0.993 | 1.190 |
| TACL N | 0.993 | 1.000 | 1296991.000 | 0.991 | 0.993 | 452130.900 |
| TG Neg | 0.992 | 1.000 | 455110.700 | 0.993 | 0.993 | 451510.300 |
| TPO Ne | 0.837 | 1.000 | 0.857 | 0.992 | 0.993 | 3648288.000 |
| HLA_A_02 | 0.718 | 1.000 | 0.852 | 0.387 | 0.993 | 0.694 |
| HLA_A_03 | 0.830 | 1.000 | 0.906 | 0.478 | 0.993 | 1.401 |
| HLA_B_35 | 0.815 | 1.000 | 1.141 | 0.806 | 0.993 | 1.144 |
| HLA_B_37 | 0.993 | 1.000 | 0.000 | 0.990 | 0.993 | 0.000 |
| HLA_B_38 | 0.068 | 1.000 | 3.229 | 0.162 | 0.988 | 4.232 |
| HLA_B_44 | 0.262 | 1.000 | 1.734 | 0.462 | 0.993 | 0.707 |
| HLA_B_45 | 0.993 | 1.000 | 0.000 | 0.092 | 0.988 | 3.503 |
| HLA_B_53 | . | . | . | . | . | . |
| HLA_B_54 | 0.994 | 1.000 | 0.000 | . | . | . |
| HLA_B_58 | 0.168 | 1.000 | 4.225 | 0.990 | 0.993 | 0.000 |
| HLA_DQB1_0201 | 0.835 | 1.000 | 0.878 | 0.855 | 0.993 | 0.904 |
| HLA_DQB1_0302 | 0.129 | 1.000 | 0.320 | 0.357 | 0.993 | 1.493 |
| HLA_DRB1_0701 | 0.680 | 1.000 | 1.241 | 0.761 | 0.993 | 1.147 |
| HLA_DRB1_15 | 0.604 | 1.000 | 1.264 | 0.464 | 0.993 | 1.391 |
| HLA_DRB1_1501 | 0.604 | 1.000 | 1.264 | 0.730 | 0.993 | 1.177 |
| Foxp3_Ae_ | 0.800 | 1.000 | 0.891 | 0.364 | 0.993 | 0.643 |
| AG49 | 0.626 | 1.000 | 1.198 | 0.496 | 0.993 | 0.800 |
| CT60 | 0.855 | 1.000 | 1.060 | 0.115 | 0.988 | 0.610 |

**Table 1B.** On-study (one month) analysis

| **Biomarkers** | **Treatment A** | | | **Treatment B** | | |
| --- | --- | --- | --- | --- | --- | --- |
|  | **P-value *** | **Adjusted P-value‡** | **Hazard**  **Ratio** | **P-value*** | **Adjusted P-value‡** | **Hazard**  **Ratio** |
| FGF_basic | 0.756 | 1.000 | 0.845 | 0.170 | 0.665 | 0.494 |
| Hu_IL2 | 0.575 | 1.000 | 0.695 | 0.131 | 0.665 | 0.444 |
| Hu_IL2R | 0.501 | 1.000 | 0.999 | **0.039** | 0.608 | 1.001 |
| Hu_IL6 | 0.276 | 1.000 | 1.000 | 0.623 | 1.000 | 1.000 |
| Hu_IL8 | 0.955 | 1.000 | 1.000 | 0.888 | 1.000 | 1.000 |
| Hu_IL10 | 0.205 | 1.000 | 2.108 | 0.488 | 1.000 | 0.639 |
| Hu_IL12 | 0.504 | 1.000 | 0.997 | 0.760 | 1.000 | 0.999 |
| Hu_IL17 | 0.313 | 1.000 | 1.758 | 0.923 | 1.000 | 1.049 |
| Hu_TNF | 0.816 | 1.000 | 1.146 | 0.929 | 1.000 | 1.047 |
| Hu_IFN | 0.718 | 1.000 | 1.219 | **0.004** | **0.156** | 0.253 |
| Hu_MIP_1a | 0.608 | 1.000 | 1.000 | 0.866 | 1.000 | 1.000 |
| Hu_MIP_1B | 0.448 | 1.000 | 1.000 | 0.814 | 1.000 | 1.000 |
| Hu_IP_10 | 0.627 | 1.000 | 1.005 | 0.822 | 1.000 | 1.000 |
| VEGF | 0.939 | 1.000 | 1.001 | 0.453 | 1.000 | 1.010 |
| Hu_IL_1B | 0.889 | 1.000 | 1.000 | 0.944 | 1.000 | 1.000 |
| IL_1_a | 0.284 | 1.000 | 1.003 | 0.341 | 1.000 | 0.997 |
| S100 | **0.031** | 0.931 | 5.967 | 0.492 | 1.000 | 0.010 |
| CRP, p | 0.673 | 1.000 | 1.000 | 0.913 | 1.000 | 1.000 |
| ANA | 0.300 | 1.000 | . | 0.750 | 1.000 | 0.721 |
| TACL N | 0.996 | 1.000 | 3699531.000 | 0.994 | 1.000 | 1291694.000 |
| TG | 0.993 | 1.000 | 456142.600 | 0.994 | 1.000 | 1257169.000 |
| TPO Ne | 0.396 | 1.000 | 0.522 | 0.630 | 1.000 | 1.642 |
| HLA_A_02 | 0.856 | 1.000 | 0.907 | 0.188 | 0.665 | 0.527 |
| HLA_A_03 | 0.298 | 1.000 | 0.533 | 0.855 | 1.000 | 1.109 |
| HLA_B_35 | 0.577 | 1.000 | 0.652 | 0.470 | 1.000 | 1.499 |
| HLA_B_37 | 0.994 | 1.000 | 0.000 | 0.994 | 1.000 | 0.000 |
| HLA_B_38 | 0.206 | 1.000 | 2.691 | 0.082 | 0.636 | 6.180 |
| HLA_B_44 | 0.067 | 0.931 | 2.921 | 0.179 | 0.665 | 0.471 |
| HLA_B_45 | 0.993 | 1.000 | 0.000 | 0.062 | 0.608 | 4.094 |
| HLA_B_53 | . | . | . | . | . | . |
| HLA_B_54 | 0.994 | 1.000 | 0.000 | . | . | . |
| HLA_B_58 | 0.070 | 0.931 | 6.863 | 0.994 | 1.000 | 0.000 |
| HLA_DQB1_0201 | 0.982 | 1.000 | 1.015 | 0.400 | 1.000 | 0.533 |
| HLA_DQB1_0302 | 0.993 | 1.000 | 0.000 | 0.357 | 1.000 | 1.543 |
| HLA_DRB1_0701 | 0.700 | 1.000 | 1.256 | 0.454 | 1.000 | 1.421 |
| HLA_DRB1_15 | 0.748 | 1.000 | 1.208 | 0.059 | 0.608 | 2.378 |
| HLA_DRB1_1501 | 0.748 | 1.000 | 1.208 | 0.141 | 0.665 | 2.000 |
| Foxp3_Ae_ | 0.929 | 1.000 | 0.955 | 0.928 | 1.000 | 0.956 |
| AG49 | 0.468 | 1.000 | 1.352 | 0.938 | 1.000 | 0.974 |
| CT60 | 0.292 | 1.000 | 1.492 | 0.176 | 0.665 | 0.634 |

*p values of the Wald test

‡False Discovery Rate (FDR) adjusted p values generated by the Benjamini and Hochberg’s procedure
