# Supplementary material for: Pro-Inflammatory Cytokines Predict Relapse-Free Survival after One Month of Interferon-α but Not Observation in Intermediate Risk Melanoma Patients
Source: PLoS One. 2015 Jul 20;10(7):e0132745. doi: 10.1371/journal.pone.0132745 (PMC4508028; doi:10.1371/journal.pone.0132745)
Supplement: S2 File — The xMAP Luminex serum assay for the selected cytokines (FGF-basic, IL-2, IL-2R, IL-6, IL-8, IL-10, IL-12p40, IL-17, TNF-α, IFN-α, MIP-1α, MIP-1β, IP-10, VEGF, IL-1β, IL-1α) was performed, utilizing baseline and one month specimens. CRP was run singly. Serum samples were tested for the presence of the following autoantibodies: antinuclear antibody screen (ANA), antithyroglobulin antibody (TG), antithyroperoxidase antibody (TPO), and anticardiolipin antibody (TACL). HLA Genotyping was conducted with Luminex/One Lambda LABTypeRSSO. PCR was used to test CTLA4 polymorphisms (AG49, CT60) and FOXP3 SNPs and microsatellites. Table A in S2 File shows the “Baseline” markers and Table B in S2 File the “On-study (one month)” markers. (DOCX) [file pone.0132745.s002.docx]

**Supplementary Table 2.** Testing of the predictive value of each marker. The xMAP Luminex serum assay for the selected cytokines (FGF-basic, IL-2, IL-2R, IL-6, IL-8, IL-10, IL-12p40, IL-17, TNF-α, IFN-α, MIP-1α, MIP-1β, IP-10, VEGF, IL-1β, IL-1α) was performed, utilizing baseline and one month specimens. CRP was run singly. Serum samples were tested for the presence of the following autoantibodies: antinuclear antibody screen (ANA), antithyroglobulin antibody (TG), antithyroperoxidase antibody (TPO), and anticardiolipin antibody (TACL). HLA Genotyping was conducted with Luminex™/One Lambda LABType™RSSO. PCR was used to test CTLA4 polymorphisms (AG49, CT60) and FOXP3 SNPs and microsatellites.

**Table 2A.** Baseline markers.

| **Interaction Term** | Estimate | | | **P-value*** |  |
| --- | --- | --- | --- | --- | --- |
| FGF_basic*trtm | | 0.39332 | 0.521 | | |
| Hu_IL2*trtm | | 1.23638 | 0.115 | | |
| Hu_IL2R*trtm | | -0.0008981 | 0.293 | | |
| Hu_IL6*trtm | | 0.0000645 | 0.675 | | |
| Hu_IL8*trtm | | -0.0000256 | 0.6566 | | |
| Hu_IL10*trtm | | 1.85646 | 0.0357 | | |
| Hu_IL12*trtm | | -0.00144 | 0.7038 | | |
| Hu_IL17*trtm | | 0.11891 | 0.8496 | | |
| Hu_TNF*trtm | | 0.19008 | 0.7698 | | |
| Hu_IFN*trtm | | 1.375 | 0.0342 | | |
| Hu_MIP_1a*trtm | | -0.0000618 | 0.6046 | | |
| Hu_MIP_1B*trtm | | -0.0000862 | 0.5388 | | |
| Hu_IP_10*trtm | | -0.01248 | 0.4443 | | |
| VEGF*trtm | | -0.01303 | 0.4604 | | |
| Hu_IL_1B*trtm | | -0.0002201 | 0.7178 | | |
| IL_1_a*trtm | | 0.00112 | 0.5962 | | |
| S100*trtm | | 1.70721 | 0.8306 | | |
| CRP__pg_mL*trt | | -3.70E-08 | 0.2453 | | |
| trtm*ANA | | 1.56792 | 0.9993 | | |
| trtm*ANA | | 15.62632 | 0.9878 | | |
| trtm*TACL | | -14.46962 | 0.9968 | | |
| trtm*TACL | | 0.1551 | 0.9999 | | |
| trtm*TG | | 0.11511 | 1 | | |
| trtm*TPO | | -14.15065 | 0.987 | | |
| HLA_A_02*trtm | | 0.27184 | 0.655 | | |
| HLA_A_03*trtm | | -0.40101 | 0.5421 | | |
| HLA_B_35*trtm | | 0.01739 | 0.9823 | | |
| HLA_B_37*trtm | | -0.06448 | 1 | | |
| HLA_B_38*trtm | | -0.46285 | 0.6997 | | |
| HLA_B_44*trtm | | 0.81073 | 0.2302 | | |
| HLA_B_45*trtm | | -13.22147 | 0.9868 | | |
| HLA_B_53*trtm | | 0 | . | | |
| HLA_B_54*trtm | | 0 | . | | |
| HLA_B_58*trtm | | 13.96034 | 0.9868 | | |
| HLA_DQB1_0201* | | -0.05706 | 0.9453 | | |
| HLA_DQB1_0302* | | -1.41607 | 0.1001 | | |
| HLA_DRB1_0701* | | -0.01193 | 0.9861 | | |
| HLA_DRB1_15*tr | | -0.05468 | 0.9316 | | |
| HLA_DRB1_1501* | | 0.11951 | 0.8547 | | |
| HLA_C_03*trtm | | -0.80081 | 0.2329 | | |
| HLA_C_06*trtm | | 0.55397 | 0.5515 | | |
| HLA_C_07*trtm | | -0.71806 | 0.2441 | | |
| HLA_C_15*trtm | | -15.21967 | 0.9873 | | |
| Foxp3_Ae_1*trt | | 0.21175 | 0.7487 | | |
| AG49*trtm | | 0.39458 | 0.4212 | | |
| CT60*trtm | | 0.57148 | 0.2007 | | |

**Table 2B.** Testing of one month markers.

| **Interaction term** | Estimate | | **P-value*** |
| --- | --- | --- | --- |
| FGF_basic*trtm | | 0.51069 | 0.4638 |
| Hu_IL2*trtm | | 0.33935 | 0.6824 |
| Hu_IL2R*trtm | | -0.00179 | 0.196 |
| Hu_IL6*trtm | | 0.0001996 | 0.5439 |
| Hu_IL8*trtm | | 8.47442E-06 | 0.9039 |
| Hu_IL10*trtm | | 1.16906 | 0.1795 |
| Hu_IL12*trtm | | -0.00225 | 0.6588 |
| Hu_IL17*trtm | | 0.50252 | 0.4946 |
| Hu_TNF*trtm | | 0.13389 | 0.8615 |
| Hu_IFN*trtm | | 1.51603 | 0.0353 |
| Hu_MIP_1a*trtm | | -0.000075 | 0.6436 |
| Hu_MIP_1B*trtm | | -0.0001328 | 0.5316 |
| Hu_IP_10*trtm | | 0.00424 | 0.6912 |
| VEGF*trtm | | -0.00839 | 0.6915 |
| Hu_IL_1B*trtm | | 0.0003125 | 0.8085 |
| IL_1_a*trtm | | 0.00589 | 0.1506 |
| S100*trtm | | 7.16879 | 0.2901 |
| CRP__pg_mL*trt | | 1.16E-08 | 0.8494 |
| trtm*ANA | | 2.44851 | 0.999 |
| trtm*ANA | | 16.00726 | 0.9876 |
| trtm*TACL | | 0.02225 | 1 |
| trtm*TACL | | 0.13753 | 1 |
| trtm*TG | | 0.11365 | 1 |
| trtm*TPO | | -1.16261 | 0.3633 |
| HLA_A_02*trtm | | 0.53293 | 0.4508 |
| HLA_A_03*trtm | | -0.65685 | 0.4219 |
| HLA_B_35*trtm | | -0.82613 | 0.383 |
| HLA_B_37*trtm | | -0.09022 | 1 |
| HLA_B_38*trtm | | -0.83615 | 0.5144 |
| HLA_B_44*trtm | | 1.6844 | 0.0322 |
| HLA_B_45*trtm | | -13.86114 | 0.9895 |
| HLA_B_53*trtm | | 0 | . |
| HLA_B_54*trtm | | 0 | . |
| HLA_B_58*trtm | | 14.78093 | 0.9883 |
| HLA_DQB1_0201* | | 0.69573 | 0.4812 |
| HLA_DQB1_0302* | | -15.89176 | 0.9881 |
| HLA_DRB1_0701* | | -0.17872 | 0.8121 |
| HLA_DRB1_15*tr | | -0.65747 | 0.3775 |
| HLA_DRB1_1501* | | -0.47967 | 0.5238 |
| HLA_C_03*trtm | | -1.05956 | 0.1817 |
| HLA_C_06*trtm | | 0.33572 | 0.7821 |
| HLA_C_07*trtm | | -0.54283 | 0.4599 |
| HLA_C_15*trtm | | -15.45736 | 0.9881 |
| Foxp3_Ae_1*trt | | -0.11809 | 0.8693 |
| AG49*trtm | | 0.32017 | 0.5469 |
| CT60*trtm | | 0.84432 | 0.0935 |

*Wald test p-values for the interaction term.
